# Dr. Davis, on Inoculations with Cow-Pox Matter

**Published:** 1799-09

**Authors:** J. F. Davis

**Affiliations:** Bath.


GENTLEMEN,

IF you think the following observations of any value, I request you to publish them.

I have the honour to be,

BATH. Your very humble Servant.

*August 6th*, 1799. J. F. DAVIS, M. D.

The extraordinary effects of darnel (*lolium temulentum*, LINN.) related by Mr. MARSH, in the fifth Number of this work, induced me to inquire whether the plant is common in our corn-fields, and whether its deleterious nature had been pointed out by any preceding writer.

The first has been satisfactorily answered by a very intelligent friend, who devotes a great part of his leisure to agricultural pursuits. He says that this species of darnel is by no means common in this neighbourhood : that he has found it in many parts of the kingdom, but always on farms occupied by negligent farmers, who were not careful to keep their land free from weeds. Several species of lolium are found in Great Britain, amongst which the *lolium perenne* and *lolium temulentum*, Linn. are most common : the former is the most abundant of all the grasses, growing in pastures and by way sides every where; the latter is more rare, growing in corn-fields only.

The powerful effects of the seeds of *lolium temulentum*, appear to have been well known. Linnæus observes, " Lolium hordeo immixtum pro cerevisia conficienda, potatores stultos et temulentos reddit."* Whence its trivial name *temulentum*. The French call it *l'yvraie*. He elsewhere says, that when made into bread with other grain, it is very injurious, if the bread be eaten hot. Haller informs us, that it occasions vertigo, intoxication, vomiting, delirium, and convulsions, which terminate in palsy ; and adds, that it produced an epidemic disease amongst some soldiers, of which many died suddenly. It kills, he says, geese, and even horses; but a small quantity mixed with their food, fattens chickens and hogs †. In the second volume of the " *Histoire de la Societé Royale de Medicine à Paris*," are some remarks on the effects of *darnel* mixed with corn in bread ; communicated to the Society by M. DE LA MAZIERE, of Poitiers. A farmer, his wife, and servant, eat bread made of the seeds of *darnel* mixed with wheat. The *darnel* was in the proportion of five to one. The two latter were seized with vomiting

* Lin. Op. Bot. cur. Gilibert, Tom. 2. p. 404.

† " *Histoire des Plantes Suisses*," Tom. 2. p. 175. Not having the Latin original, I have quoted the French Translation, published at Berne, 1791.

vomiting and purging, and refufed to eat any more of the bread. The farmer continued to ufe it the three following days, and died after fuffering the moft fevere colic pains. I cannot learn, however, that any perfon has noticed the peculiar affection of the calves of the legs defcribed by Mr. Marfh.

Externally applied, darnel is faid to produce anodyne properties, and therefore is recommended by CELSUS in fractures of the ribs: "Gravioribus vero doloribus, urgentibus, cataplafma imponi quoque conveniet, vel ex lolio vel ex hordeo, cui pinguis fici tertia pars fit adjecta*." ARETÆUS adminiftered it in pleurify; GALEN applied it, mixed with vinegar, to wounds.

---

P. S. The fuccefsful practice of inoculating with cow-pox matter, of which your valuable Journal has been the vehicle, gives me great fatisfaction. I am fearful, however, that fome obfervations of mine, communicated to my friends not long fince, may have induced them to entertain an unfavourable opinion of it. If fo, what follows will, I hope, remove it entirely.

In March, two children (COOMBES and JAMES), were inoculated under my direction, with vaccine matter fent from London by Dr. PEARSON. The punctures inflamed in both children, and formed tumours; but Coombes's only advanced to a ftate of vefication, James's vanifhed about the eighth day. Coombes was very feverifh and drowfy on the tenth day; while James did not appear to have been at all indifpofed. On the twelfth an eruption appeared on different parts of the bodies of both children; two or three puftules fuppurated on that of Coombes, but they difappeared on James in a day or two, fome indeed in a fhorter time. I thought, notwithftanding, that he had undergone the vaccine difeafe. On applying the teft of variolous matter he became infected, and had many puftules. Coombes, on the contrary, refifted inoculation for the variolous difeafe, and frequently played with James during its progrefs, without taking it.

Through the friendly affiftance of Dr. WOODVILLE, I have fince feen a confiderable number of patients labouring under cow-pox; and am in confequence fully convinced, that the inflammation was infufficie nt to prove that James underwent the vaccine difeafe, and that the eruption was merely adventitious.

---

### *To the Editors of the Medical and Phyfical Journal.*

GENTLEMEN,

THE following communication I have received from Mr. BARLOW, Surgeon, at Bolton.

As

---

* Aur. Corn. Cels. lib. viii. cap. 9.

